# Implication of Membrane Androgen Receptor (ZIP9) in Cell Senescence in Regressed Testes of the Bank Vole

**DOI:** 10.3390/ijms21186888

**Published:** 2020-09-19

**Authors:** Magdalena Profaska-Szymik, Anna Galuszka, Anna J. Korzekwa, Anna Hejmej, Ewelina Gorowska-Wojtowicz, Piotr Pawlicki, Małgorzata Kotula-Balak, Kazimierz Tarasiuk, Ryszard Tuz

**Affiliations:** 1 University Centre of Veterinary Medicine JU-UA, University of Agriculture in Krakow, Mickiewicza 24/28, 30-059 Krakow, Poland; magdalena.profaska-szymik@urk.edu.pl (M.P.-S.); anna.galuszka@urk.edu.pl (A.G.); piotr.pawlicki@urk.edu.pl (P.P.); kazimierz.tarasiuk@urk.edu.pl (K.T.); 2 Department of Biodiversity Protection, Institute of Animal Reproduction and Food Research of Polish Academy of Sciences, Tuwima 10, 10-748 Olsztyn, Poland; a.korzekwa@pan.olsztyn.pl; 3 Department of Endocrinology, Institute of Zoology and Biomedical Research, Jagiellonian University in Krakow, Gronostajowa 9, 30-387 Krakow, Poland; anna.hejmej@uj.edu.pl (A.H.); ewelina.gorowska@uj.edu.pl (E.G.-W.); 4 Department of Genetics, Animal Breeding and Ethology, Faculty of Animal Science, University of Agriculture in Krakow, Mickiewicza 24/28, 30-059 Krakow, Poland; rztuz@cyf-kr.edu.pl

**Keywords:** androgen, bank vole, senescence, testes

## Abstract

Here, we studied the impact of exposure to short daylight conditions on the expression of senescence marker (p16), membrane androgen receptor (ZIP9) and extracellular signal-regulated kinase (ERK 1/2), as well as cyclic AMP (cAMP) and testosterone levels in the testes of mature bank voles. Animals were assigned to groups based on an analysis of testis diameter, weight, seminiferous tubule diameter and the interstitial tissue area: group 1, not fully regressed (the highest parameters); group 2 (medium parameters); or group 3, regressed (the lowest parameters). Cells positive for p16 were observed only in the seminiferous tubule epithelium. However, in groups 1 and 2, these were mostly cells sloughed into the tubule lumen. In group 3, senescent cells resided in between cells of the seminiferous epithelium. Staining for ZIP9 was found in Sertoli cells. Western blot analysis showed a trend towards a decreased expression of p16 and ZIP9 in the testes of the voles in groups 2 and 3, compared to group 1. In addition, a trend towards an increased expression of ERK, as well as an increase of cAMP and testosterone levels, was revealed in group 2. In the regressed testes, a functional link exists between senescence and androgen levels with implication of ZIP9 and cAMP/ERK signaling pathways.

## 1. Introduction

Seasonal breeding describes a lack of continual reproduction throughout the year due to circannual fluctuations in environmental conditions resulting from climatic seasons in particular species or populations inhabiting nonequatorial areas. This is an adaptive process that ensures that new individuals are born and grow up during the season that provides the best conditions for survival. In males of various species, a reduction in the size of the testes accompanied by lower androgen production, spermatogenesis arrest and inhibited mating behavior has been observed [1,2,3,4,5,6]. In these animals, the hypogonadal axis is modulated through pronounced involvement of melatonin and kisspeptins, and thus, reproduction is activated during the breeding season and halted during the resting period, impacting both spermatogenesis and sex steroid synthesis [7]. It should be noted that in some mammals, hyperthermia, bone loss, disruption of sleep patterns and oxidative stress serves as energy for reproduction [8]. However, data on the molecular mechanisms of reproductive system quiescence remain incomplete.

In promiscuous bank voles, a common rodent species in Europe and Asia, the reproductive system atrophy takes place between late October and late March, and is associated with marked alterations in the testes e.g., depletion of the spermatogenic cells, cell apoptosis and proliferation [9,10]. The subsequent shrinkage in the size of the testes is common in the males of most seasonally breeding species. Only in a few species do the inactive testes maintain some meiotic active cells and meiosis onset is not completely interrupted [11]. Moreover, a reduction in the size of Leydig cells has been reported for most seasonal breeders [12,13,14,15]. This hypotrophy is generally associated with a reduction in the volume of smooth endoplasmic reticulum. According to Seco-Rovira et al. [16], the lowering of androgen levels is not attributed to changes in cell ultrastructure. Rather, a wave of cell death during the regression period and then proliferation during the recovery period are implicated in cyclic variations of Leydig cell number and function. Apoptotic germ cells are phagocytized by Sertoli cells, i.e., somatic cells of the seminiferous epithelium. On the other hand, in some species, apoptosis does not cause a massive germ cell depletion during testes regression [17]. Interestingly, in the mole, a new mechanism of testes regression has been reported based on massive desquamation (sloughing, exfoliation) of live, nonapoptotic meiotic and postmeiotic germ cells [18]. In this species, there is an indication that testes regression is regulated by modulation of the expression and/or distribution of the cell adhesion (junctional) molecules connecting Sertoli and germ cells in the seminiferous epithelium with constant involvement of low intratesticular androgen levels.

The potency of the negative feedback actions of testosterone is one of the principal mechanisms driving seasonal changes in reproductive system function [19,20]. Findings, both in vitro and in vivo, in prostate cancer cells revealed that androgen deprivation induces senescence, a stress response that stops the proliferation of dysfunctional cells [21]. These cells remain metabolically active and have heightened survival, which makes them highly viable and resistant to apoptosis [22]. Moreover, androgen activation of intracellular signaling pathways via increase of cAMP, activation of extracellular signal-activated kinase 1/2 (ERK1/2) and activation of transcription factors were recently demonstrated in the developing testes [23]. Cell membrane ZRT and Irt-like Protein 9 (ZIP9), belonging to zinc transporters, were shown to be involved in these quick signal transduction pathways. To date, only initial efforts have been undertaken to understand the role of membrane androgen receptor ZIP9 in the male gonad. Historically, it has been generally accepted that the nuclear androgen receptor expressed by testicular somatic cells is a crucial component of androgen signaling [24].

Bank voles offer a unique experimental model. They are maintained through the inbreeding system in a few laboratories worldwide. Dynamic seasonal changes in their reproductive physiology are regulated by artificial light adjustment without temperature and food restrictions [25]. Thus, subtle reproductive organ atrophy occurs in voles exposed to short light conditions. These animals can reproduce but with lower frequency when compared to long light-regulated ones. Studies performed over many years by our team have clearly shown that the bank vole is a useful model to study mechanisms relying upon disturbances of male reproductive function due to imbalances of sex hormones [26,27,28,29,30,31]. In light of the above facts, the role of ZIP9 in relation to senescence in the responses of regressed bank vole testicular tissue is fascinating.

## 2. Results

### 2.1. Weight, Diameter, Morphometry and Morphology of Bank Vole Regressed Testes

**Figure 1:**
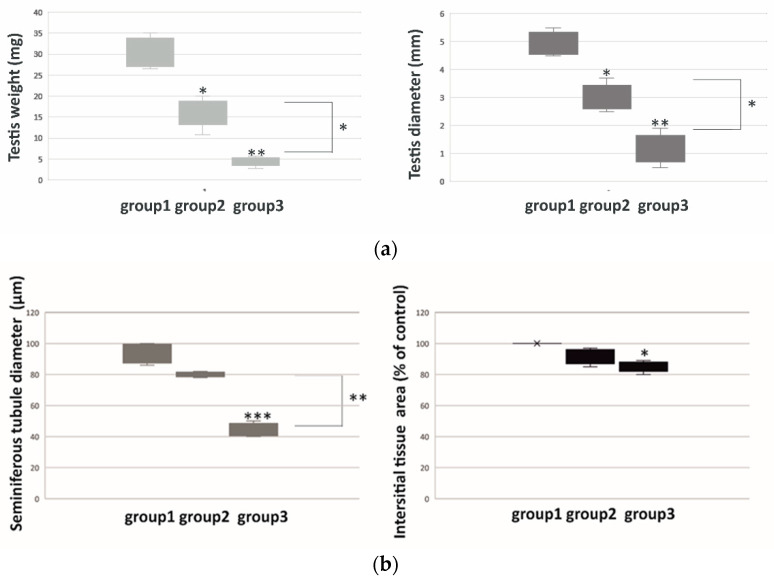
Weight and diameter (**a**) and seminiferous tubule diameter and interstitial tissue area (**b**) of regressed testes of bank voles. Boxplots represent medians ± quartile range. Significant differences between groups 1, 2 and 3 are denoted as ^∗^ *p* < 0.05, ^∗∗^ *p* <0.01 and ^∗∗∗^ *p* < 0.001. Group 1 serves as a control. Analysis was performed in triplicate.

Bank voles were divided into three groups according to diameter and weight of their testes, as well as the seminiferous tubule diameter and interstitial tissue area measurements (Figure 1). In detail, when compared to group 1 (voles with the highest testes parameters), voles in group 2 had medium size (*p* < 0.05) and weight (*p* < 0.05), while voles in group 3 had the lowest size (*p* < 0.01) and weight (*p* < 0.01) of the testes (Figure 1a).

Morphometric analyses revealed no differences in seminiferous tubule diameter and interstitial tissue area between the voles of group 1 and 2 (Figure 1b). In contrast, marked differences in tubule diameter and interstitial tissue area were found in the voles of group 3, when compared to group 1 (*p* < 0.001) and group 2 (*p* < 0.01), and when interstitial tissue area (*p* < 0.05) was compared to group 1.

**Figure 2:**
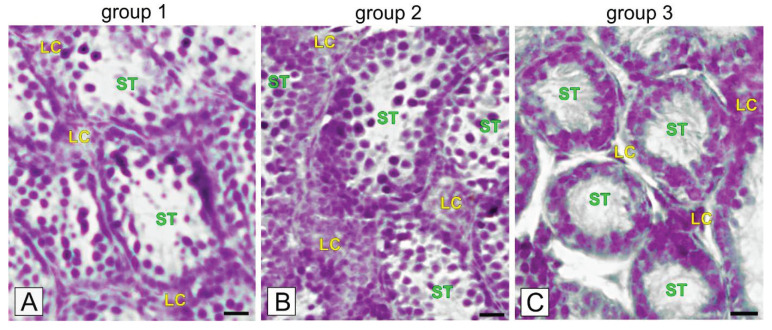
Representative microphotographs of morphology of bank vole testes with different stages of their regression (**A**; group 1 with the lowest regression); (**B**; group 2 with medium regression) and (**C**; group 3 the highest regression). Hematoxylin-eosin staining. Bar 45 µm. Staining was performed at three serial sections from each animal. LC-Leydig cells; ST-seminiferous tubules.

Histological staining of testes sections of group 1 voles revealed that half of the seminiferous tubules were lined mainly with a single layer of cells: spermatogonia, a few spermatocytes and Sertoli cells (Figure 2A).

Of note, cells were not tightly adhered and some cells were visibly sloughed into the large tubule lumen. In the testes sections of group 2 voles, there were two to three layers: spermatogonia, spermatocytes. Sertoli cells were observed in most of the tubules with single cells exfoliated into the lumen (Figure 2B). In the testes of group 3 voles, a few spermatogonia and Sertoli cells were observed (Figure 2C). No sloughed cells were visible in the lumen. Interstitial cells were located in small or midsized groups in between the seminiferous tubules. In the voles of group 3, interstitial tissue cells were always located in small groups.

### 2.2. Localization of p16 and ZIP9 Receptor in Bank Vole Regressed Testes

**Figure 3:**
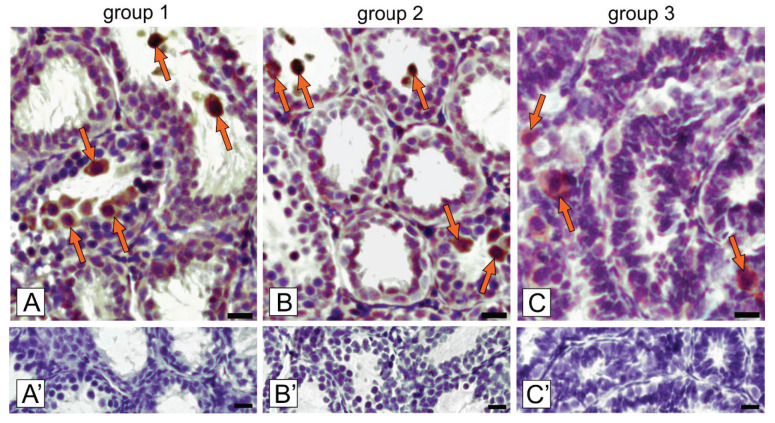
Representative microphotographs of immunohistochemical localization of p16 in bank vole testes (**A**; group 1 with the lowest regression, **B**; group 2 with medium regression; and **C**; group 3 with the highest regression). Staining with DAB and counterstaining with hematoxylin. Bar 45 µm. Staining was performed at three serial sections from each animal. Arrows depict senescent germ cells (group 1 and 2) and germ/Sertoli cells (group 3). **A’**–**C’**—negative controls.

In voles of all examined groups, senescent cells were occasionally visible only in seminiferous tubules (Figure 3A–C).

In detail, in group 1 senescent cells (25.0 ± 1.3%) were exfoliated to the lumen and only single-adhered senescent cells were visible (Figure 3A). Meanwhile, in group 2, senescent cells (18.06 ± 2.3%; *p* < 0.01) were found as either exfoliated or adhered to the tubule epithelium (Figure 3B). In group 3, a few senescent cells (7.33 ± 0.9%; *p* < 0.001) were tightly adhered to the cells of the tubule epithelium (Figure 3C). Negative controls showed no positive staining (Figure 3A’–C’).

**Figure 4:**
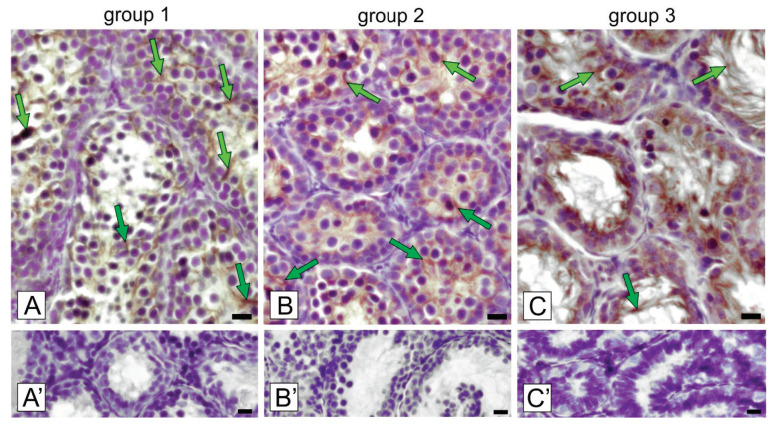
Representative microphotographs of immunohistochemical localization of ZIP9 in bank vole testes (**A**; group 1 with the lowest regression; **B**; group 2 with medium regression; and **C**; group 3 with the highest regression). Staining with DAB and counterstaining with hematoxylin. Bar 45 µm. Staining was performed at three serial sections from each animal. Arrows depict Sertoli cells with positive staining. **A’**–**C’**—negative controls.

In all groups of voles with regressed testes, cytoplasmic localization of ZIP9 was revealed (Figure 4A–C).

The positive signal was exclusively visible in Sertoli cells with the strongest expression in regressed testes of voles of group 3. Negative controls showed no positive staining (Figure 4A’–C’).

### 2.3. Expression of p16, ZIP9 and ERK1/2 in Bank Vole Regressed Testes

**Figure 5:**
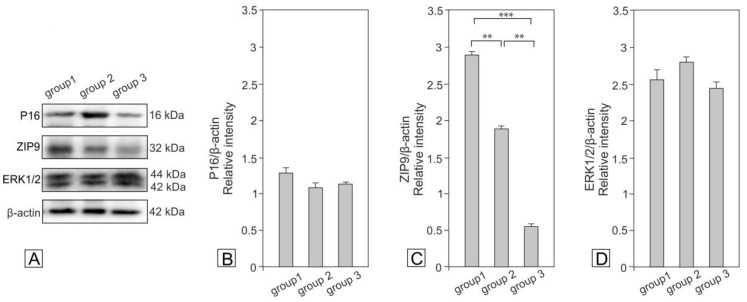
Representative blots (**A**) of qualitative expression and relative expression (arbitrary units) of p16 (**B**), ZIP9 (**C**) and ERK1/2 (**D**) in regressed bank vole testes. The relative amount of respective proteins normalized to β-actin. Relative intensity of bands from three separate analyses is expressed as means ± SD. Asterisks show significant differences between the groups 1, 2 and 3. Significant differences are denoted as ^∗∗^ *p* <0.01 and ^∗∗∗^ *p* < 0.001.

Expression of p16 showed a decreasing trend in the testes of animals of groups 2 and 3 compared to those of group 1 (Figure 5).

The expression of ZIP9 decreased significantly in the testes of animals in groups 2 (*p* < 0.01) and 3 (*p* < 0.001). Interestingly, the expression of ERK1/2 showed different changes in the testes of voles in groups 2 and 3 when compared to group 1; however, they were not significant.

### 2.4. Concentration of cAMP and Testosterone in Bank Vole Regressed Testes

In the testes of group 1 voles, cAMP level was 13.04 ± 1.3 pmol/mL, while in group 2 it was 15.98 ± 1.51 pmol/mL (*p* < 0.01). The lowest cAMP concentration was revealed in the voles of group 3 (10.45 ± 0.97 pmol/mL) (*p* < 0.05).

Similarly, the highest decrease in testosterone level was revealed in testes of group 3 voles, i.e., 21.09 ± 1.11 ng/mL (*p* < 0.001), whereas it was 27.43 ± 2.88 ng/mL in group 1 and 29.57 ± 3.05 ng/mL in group 2 (*p* < 0.05).

## 3. Discussion

This study shows, for the first time, that senescent cells are present in the spermatogenic epithelium in various stages of bank vole testes regression. Over time, complete suppression of spermatogenesis and a precipitous decline in testicular weight have also been reported in other long day breeding rodents [32]. Transfer of the animals to short photoperiod or blinding them both resulted in atrophic changes (in tubules and interstitium) and changes in biochemical status of the testes, as were found here in wild bank voles. Gravis [33] reported that in such animals, Leydig cell size was decreased, lipid droplets were absent and the size of Golgi complex and endoplasmic reticulum were reduced. These changes result in severely suppressed sex steroid production which is considered a primary stress factor. Such androgen deficiency is also mirrored in an atrophy of the accessory organs of the male reproductive system [27,34].

Herein, we reveal that mature voles collected in the same short daylight season time and from the same geographic region show various stages of reproductive system atrophy. This atrophy can be linked to exposure to sunlight, type and availability of food and interactions with other males/females, all of which have an effect on the individual vole’s sex hormonal status [35,36]. Regression of the testes can be complete or nearly complete if, during the exposure to a short photoperiod, laboratory animals are treated with combinations of prolactin and gonadotropin-releasing hormone [37,38,39]. An accompanying testes quiescence status in examined voles with various stages of regression, were differences found in hormonal and signaling molecule expression/concentrations.

Senescence limits the proliferation of aged or damaged cells, with such aging hallmarks as: (i) primary, i.e., the effects of age-associated damage; (ii) antagonistic, i.e., responses to damage; and (iii) integrative, i.e., the consequences of the responses and effects of the aging phenotype [40]. All possibilities can take place in regressed vole testes. It is likely that in animals undergoing reproduction quiescence, physical factor (short light) and cell endogenous factors (levels of signaling molecules and their cross-talks) have an impact on the atrophic tissue microenvironment. Thus, senescence may be a stress response to the above factors, triggered by insults associated with precocious aging (genomic instability and telomere attrition). There is also an intimate link between senescence and other antagonistic hallmarks of aging. For example, senescent cells display decreased mitophagy. This results in a defective mitochondrial network that may contribute to metabolic dysfunction [41]. It is also possible that similar to the attenuated Leydig cells of regressed testes mitochondria, aging takes place in spermatogenic cells, as was reported in energy demanding processes occurring during pathological cell conditions [42]. In addition to decreased testosterone levels, decreased cAMP levels can perturb mitochondria function and its interaction with other cytoplasmic molecules [41]. Consequently, abnormally configured spermatogenic cells are unable to progress to spermatozoa and are sloughed or degenerate [43].

We found here that senescent cells were present in both incompletely and completely regressed bank vole testes. However, their number and cell type undergoing senescence were distinct. In completely regressed animals, spermatogenic cells were senescent, but this can also be true for single Sertoli cells. Spermatogenic cell senescence may be also related to functional crosstalk of spermatogenic cells with Sertoli cells. Here, we found differences in the interstitial tissue area between incompletely regressed and regressed testes. This may be directly linked with a decreased Leydig cell number, as we demonstrated in our prior study in short day bank voles [31]. It is also well-known that mammalian Leydig cells exhibit various levels of steroidogenic activity due to Leydig cell population heterogeneity [44]. Moreover, any imbalance in sex steroid levels results in interstitial tissue alterations [45,46]. The changes in the interstitial tissue significantly affect seminiferous tubules [37,38,39,40,41,42,43,44,45,46,47,48,49,50]. For instance, in patients with Klinefelter’s syndrome (XXY), lowering the level of testosterone leads to gradual spermatogenesis destruction. This results in fibrosis of the seminiferous tubules and hyperplasia of the interstitial tissue [51]. Analysis of the expression of cell cycle protein p16 by western blot in regressed testes revealed a tendency towards p16 decrease when compared to incompletely regressed testes. This clearly indicates that the highest number of senescent cells is present when regression has started. Of note, no senescent or singular senescent cells were revealed in the testes of inbred bank voles that were actively reproducing (unpublished data).

Independent of the regression status of vole testes, ZIP9 is expressed exclusively in Sertoli cells. This is in line with studies in mouse and rat Sertoli cells [52,53]. Thus, decreased expression of ZIP9 suggests decreasing Sertoli cell numbers during regression, as was recently shown in Syrian hamsters [54]. In Sertoli cells (TM4), the ZIP9 signal detected outside of the Sertoli cell nucleus and in the cell membrane was enhanced by the addition of testosterone or by Notch signaling inhibition, especially in perinuclear region [52]. Plasma membrane distribution of ZIP9 in breast cancer cells (MDA-MB-468) and prostate cancer cells (PC3) has been linked to its androgen binding [55]. Findings on the role of ZIP9 in the regulation of zinc homeostasis in secretory pathways was demonstrated in cervical cancer cells [56]. Zinc and iron are not only components of many proteins, such as transcription factors and metalloenzymes, but also play a role as second messenger molecules [57,58]. Therefore, precise control of ion concentration seems to be important in physiological testes regression and cell senescence relative to low androgen levels. Recently, we reported that hormonal disturbances associated with cryptorchidism or germ cell tumors in dog testes are linked with increased senescent spermatogenic cell number [59]. Similarly, we found increased numbers of senescent spermatogenic cells in inbred rabbit, nutria and chinchilla seminiferous tubules with spermatogenic alterations [60]. After hormonal castration in dogs, decreased ZIP9 expression was revealed [55]. This is in partial agreement with the results obtained herein, especially in voles in group 2. Specifically, trends towards a decreased expression of ZIP9, together with subtle increases in androgen levels, were revealed. Concomitantly, in that group, ERK showed a trend towards increased expression, and cAMP levels were slightly increased. This suggests potential for many other breakthrough events at the cellular (e.g., autophagy), hormonal (e.g., via prolactin regulation) and molecular levels (ubiquitin-proteasome machinery activity) that take place between initiation of regression and its completion [61,62]. In bank voles, decreased cAMP and androgen production coincided with a decreased number of senescence cells and ZIP9 expression, particularly in bank voles in group 1 (early regression) and group 3 (completed regression). In Sertoli cells (TM4), the presence of testosterone resulted in both ZIP and androgen receptor inhibition [42]. Of note, recently, Bulldan et al. [21] demonstrated that the blockade of ZIP9 in androgen receptor-deficient rat Sertoli cells leads to perturbations in the expression of tight junction proteins and suggests a role of ZIP9 in blood-testes-barrier stability. Therefore, the observed exfoliation of seminiferous tubule senescent cells can be linked with decreased androgen level, perturbations in ZIP9 expression and/or interactions of ZIP9 with various signaling molecules.

Our results are in line with those showing the involvement of cAMP in ZIP9 signaling, as previously demonstrated in ovarian and prostate cells where cAMP responses to androgen stimulation were dependent of the cell phenotype. Activation of ZIP9 signaled by testosterone results in increased cAMP production in ovarian follicle cells of the Atlantic croaker. The opposite effect was found in ZIP9-transfected human prostate cancer (PC-3) cells, where testosterone decreased cAMP concentration [55,63]. Presumably, cAMP impacts, at least in part, both senescence and ZIP9 signaling during testes regression. On the other hand, results by Chen [64] showed a marked decrement in the expression of CREB with senescence. The diminished expression of CREB may contribute to altered cAMP-mediated regulation of gene expression with senescence. In brain cells, alterations in senescence-related cAMP/CREB signaling are common during aging [65]. According to our findings, cAMP level alterations during testes regression are concomitant with testicular cell senescence, together with dynamic changes in ERK expression and testosterone levels. The messenger for extracellular and intracellular signals, ERK1/2 pathway, plays a vital role in many cellular processes including senescence [66]. Studies demonstrated that ERK1/2 induces cell senescence in breast, ovary cancer, murine cerebral neurons and melanoma cells [67,68,69]. Our prior studies showed that ERK1/2 seems to be directly and/or indirectly involved in cellular senescence in dog pathological testes [59]. Here, in regressed testes of bank voles, ERK acts as a copartner of cAMP in the regulation of senescence under a testosterone control.

With reference to this result, we suggest that androgens drive senescence via ZIP9 in regressed vole testes. Based on the results from our recent studies in canine tumor testes, and Rodentia and Lagomorpha inbreeds, a strong correlation between sex steroid levels and increased senescence exists [59,60]. In human prostate cancer, supraphysiological androgen levels induce senescence [70]. Recent findings by Chatterjee et al. [71] confirmed that elevated androgen levels play a protective role. The present study is in agreement with the findings of Wang et al. [72], demonstrating potential implications of senescence along with aging in declining male fertility. Episode-like pulse testosterone supplementation therapy induces tumor senescence and growth arrest down-modulating androgen receptors through its effect on ERK1/2 signaling [73]. Interestingly, recent studies by Schmidt et al. [74] reported that cellular senescence in human testicular peritubular-myoid cells is not associated with a decreased expression of crucial genes (contractility markers and androgen receptor). However, striking morphological changes in these cells are accompanied by altered cellular protein levels.

**Figure 6:**
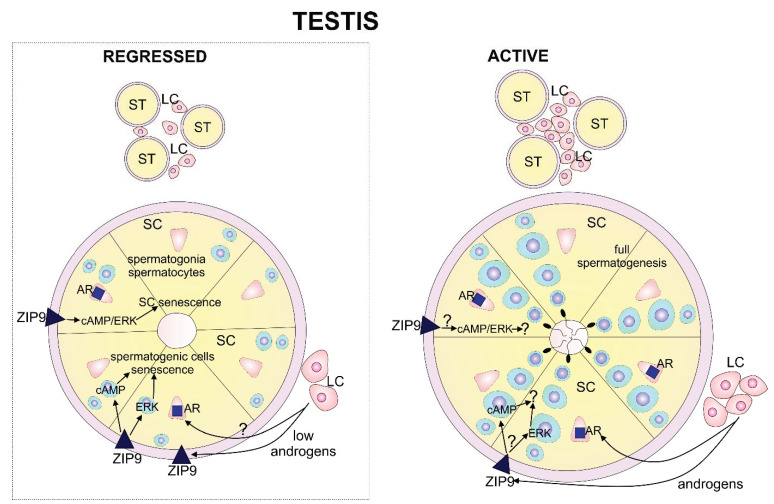
Schematic representation of the local effects of androgens in regressed bank vole testes, together with hypothetical events and interactions in active testes (not studied here). Interactions of low androgen with ZIP9, ERK (ERK1/2) and cAMP result in germ cell senescence in regressed testes. Membrane-located androgen receptor (ZIP9) is activated in Sertoli cells due to low levels of androgens. Upon androgen binding to ZIP9, cAMP and ERK in both Sertoli cells (nutritional and mechanical function) and germ cells are activated. Possibly, these interactions are also influenced by crosstalk between these two types of cells. In the active testes, proper androgen levels activate ZIP9 and androgen receptors (AR). AR seems to be dominant in its response via gene regulation that maintains spermatogenic and steroidogenic testes function. Putative interactions are marked with “?”. LC-Leydig cells; SC-Sertoli cells.

Summing up, the present study has identified senescence and ZIP9 signaling pathways as regulators of spermatogenesis in regressed bank vole testes. The decreased expression of ZIP9 in senescence cells, as well as dynamic changes in ERK1/2 and cAMP messengers vital for basic functions of spermatogenic epithelium, are consistent with these findings. The regulation of the quiescence of spermatogenic cells and spermatogenesis renewal in regressed testes are under continuous influence of low testosterone levels, which act via ZIP9 and then ERK/cAMP (Figure 6).

Therefore ZIP9 signaling also may be considered an important pathway controlling regressed vole testes function. This contributes to the senescence of spermatogenic epithelium cells in response to low androgens, and is under the control of exogenous physical and endogenous molecular regulations. Given the occurrence of senescence and ZIP9 signaling in regressed testes, searching for the factors and mechanisms interfered in nonreproductively active testes is crucial to understanding the physiology and pathology of spermatogenesis. Therefore, it would be worthwhile focusing further research on the ways in which senescence and nonclassical androgen signaling integrate with ERK and cAMP signaling in the seminiferous epithelium in early, medium and full regression testes conditions.

## 4. Materials and Methods

### 4.1. Animals

Mature bank vole males (2–3 month old) were sorted into three groups according to their testes regression status [size (diameter measured in the middle of the testes by medical caliper) and weight]: not fully regressed, early regression (group 1—voles with the largest morphometric testes parameters, *n* = 5); not fully regressed, medium regression (group 2—voles with medium morphometric testes parameters, *n* = 7), and fully regressed (group 3—voles with the lowest morphometric testes parameters; *n* = 5). The age of each vole was assigned according to the root length of the first lower molar [75]. Seriously injured or freshly dead animals (due to agricultural field activity) were collected in the fields near Popielanski Forest (Popielno, Poland) in late October 2019. The testes were dissected and, after measurement of size and weight, one testis was immediately snap-frozen in liquid nitrogen. The other one was fixed in 4% paraformaldehyde. The study (no. 46/2015/D016/2020) conformed to the Institutional Animal Care and Use Committee guidelines of the University of Agriculture in Krakow.

### 4.2. Morphometry

Hematoxyline and eosine (H-E) staining was performed on 5-μm paraffin-removed sections. The diameter of the seminiferous tubules was measured at ×100 magnification using ImageJ software; https://imagej.nih.gov/ij/docs/intro.html. On average, 60 circular tubules were measured per slide. When the tubular sections were slightly oval, only the smaller diameter was measured. Mean was determined for each animal and data (means ± SD) were expressed in μm. The area of the interstitium occupied by Leydig cells was calculated from ImageJ measurements of freehand outlines drawn along the circumference of interstitial cell clusters. The area of Leydig cells was determined at ×400 magnification in 40 random fields of vision for each section examined, and was expressed as a percentage of the area obtained from control (group 1) calculations ± SD. For the control group, area was adopted as 100%.

### 4.3. Western Blot Analysis

The proteins were extracted from testicular tissue with a cold RIPA buffer (Thermo Scientific, Waltham, MA, USA) supplemented with protease inhibitors (Sigma-Aldrich, St. Louis, MO, USA). Separation of protein preparations by SDS-PAGE under reducing conditions and transfer of proteins to polyvinylidene difluoride membranes were performed as described before [66]. Nonspecific binding sites were blocked with a solution of 5% (*wt/v*) nonfat dry milk containing 0.1% (*v/v*) Tween^®^ 20 (Sigma-Aldrich, St. Louis, Missoury USA). Next, the membranes were incubated with the respective primary antibody (Table 1) at 4 °C overnight, followed by a horseradish peroxidase-conjugated secondary antibody (1:3000; Vector Laboratories, Burlingame, CA, USA) for 1 h at room temperature.

Proteins were detected by chemiluminescence, and documented with a ChemiDocTM XRS+ System (Bio–Rad Laboratories, Hercules, CA, USA). Specificity of antibodies was assessed with the use of blocking peptide and/or positive control. All immunoblots were stripped and reprobed with an anti-β-actin antibody as the loading control. The molecular weights of target proteins were estimated by reference to standard proteins (Sigma–Aldrich, St. Louis, Missoury USA). To obtain quantitative results, immunoblots were analyzed densitometrically using the ImageLab software (Bio–Rad Laboratories, Hercules, CA, USA). Each data point was normalized against its corresponding actin data point.

### 4.4. Immunohistochemistry

Immunohistochemical staining was performed on 5-μm serial sections of testicular tissue. Antigen retrieval, endogenous peroxidase neutralization and blocking of nonspecific binding sites were performed as described previously [76]. Thereafter, the sections were incubated overnight at 4 °C with a primary antibody (Table 1). On the next day, a biotinylated secondary antibodies (1:400; Vector Laboratories, Burlingame, CA, USA) were applied for 60 min. The staining was developed with an avidin-biotinylated horseradish peroxidase complex solution (1:100; Vectastain Elite ABC Reagent, Vector Laboratories, Burlingame, CA, USA) for 30 min, followed by 0.05% 3.3′-diaminobenzidine tetrachloride containing 0.01% (*v/v*) H_2_O_2_ and 0.07% (*wt/v*) imidazole. Sections were counterstained with Mayer’s hematoxylin. Negative controls in the absence of primary antibodies were performed for each immunostaining. Sections were examined with a Nikon Eclipse Ni microscope (Nikon Instech Co., Ltd., Tokyo, Japan).

Senescent cells were observed using a 40 × objective, and scored by an observer blinded. For each testicular tissue, senescent cells in cross-sectional profiles (∼100) were counted and expressed as mean ± SD (senescent cells/testes).

### 4.5. Measurement of cAMP Concentration

The amount of cAMP produced by regressed testes was determined by DRG Cyclic AMP direct ELISA (DRG, Inc. Int. Springfield, NJ, USA) according to the manufacturer’s instructions. Testicular homogenates were run in duplicate. Optical density was measured with microtiter platereader at 450 nm. The sensitivity of the assay was 0.64 pmol/mL.

### 4.6. Measurement of Testosterone Level

Testosterone Enzyme Immunoassay Kit (DRG, Inc. Int. Springfield, New Jersey, USA) was used for measurement of hormone content in testicular homogenates, each in duplicate, according to the manufacturer’s instructions. The absorbance was measured by microtiter platereader at λ = 450 nm. The sensitivity of the assay was 0.083 ng/mL.

### 4.7. Statistics

Each variable was tested using the Shapiro-Wilk W-test for normality. Homogeneity of variance was assessed with Levene’s test. Since the distribution of the variables was normal and the values were homogeneous in variance, all statistical analyses were performed using one-way analysis of variance (ANOVA) followed by Tukey’s post hoc comparison test to determine which values differed significantly from the controls. The analysis was made using Statistica software (StatSoft, Tulsa, OK, USA). Data were presented as mean ± SD or median ± quartile range. Data were considered statistically significant at *p* < 0.05. All the experimental measurements were performed in duplicate or triplicate from material derived from different animals.

## Figures and Tables

**Table 1:** Primary antibodies used for western blot and immunohistochemistry.

Antibody	Host Species	Vendor	Dilution
ZIP9	Rabbit	Sigma-Aldrich cat.no. SAB3500599	1:200 (IHC) 1:1500 (WB)
p16	Rabbit	Abcam cat.no. ab151303	1:50 (IHC) 1:1000 (WB)
γH2AX	Mouse	Abcam cat.no. ab11174	1:250 (IHC) 1:1000 (WB)
ERK ½	rabbit	Abcam cat. no. ab17942	1:1500 (WB)
β-actin	Mouse	Sigma–Aldrich cat. no. A2228	1:3000 (WB)

Abbreviations: ZIP (ZRT, IRT-like protein; membrane androgen receptor); p16 (cell cycle protein); γH2AX (phosphorylated H2AX histone); extracellular signal—Regulated kinase 1/2 (ERK1/2); beta actin (β-actin). Antibodies suppliers: Sigma–Aldrich, St. Louis, Missoury USA. Abcam, Cambridge, MA, USA.
